# Supplementary material for: Structural basis for potent neutralization of SARS-CoV-2 and role of antibody affinity maturation
Source: Nat Commun. 2020 Oct 27;11:5413. doi: 10.1038/s41467-020-19231-9 (PMC7591918; doi:10.1038/s41467-020-19231-9)
Supplement: Supplementary file 1 — Supplementary Information [file 41467_2020_19231_MOESM1_ESM.pdf]

Supplementary Information for

Structural basis for potent neutralization of SARS-CoV-2 and role of antibody affinity maturation

Hurlburt et al.

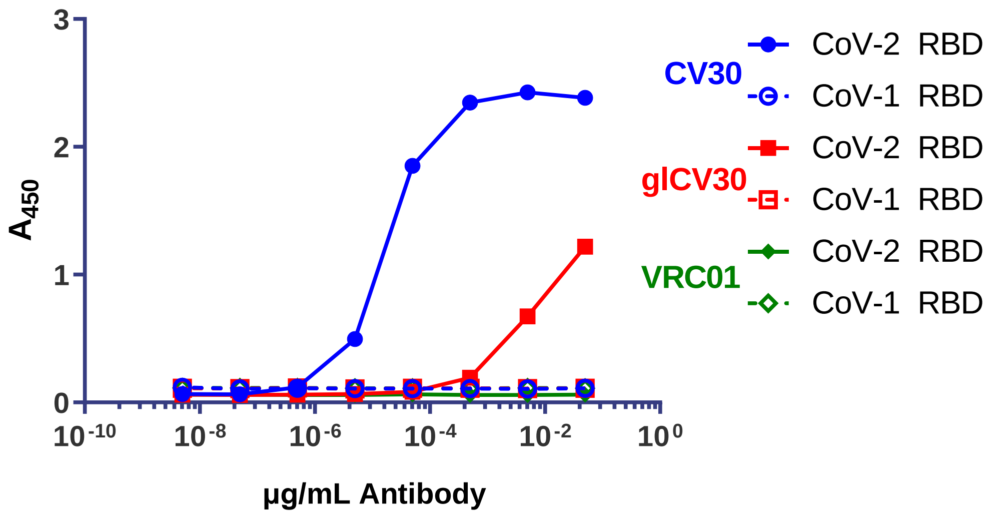

Supplementary Figure 1, related to Figure 1. ELISA of CV30 and glCV30 against the RBD from SARS-CoV-1 and SARS-CoV-2. CV30 is specific to SARS-CoV-2. CV30 is shown in blue, glCV30 is shown in red, and VRC01, an anti-HIV control antibody, is shown in green. The SARS-CoV-1 and SARS-CoV-2 RBD data is shown with an open or closed symbol. The points are an average of two replicates. Source data are provided as a Source Data file.

```
CV30_Heavy      EVQLVESGGGLIQPGGSLRLSCAASGVIVSSNYMSWVRQAPGKGLEWVSVIYSGGSTY  58
                                         ▪▪▪ ▪▪▪▪                  ▪▪▪ ▪ ▪
B38_Heavy       EVQLVESGGGLVQPGGSLRLSCAASGFIVSSNYMSWVRQAPGKGLEWVSVIYSGGSTY  58
                                         ▪▪▪ ▪▪▪▪                  ▪▪▪ ▪▪▪
CB6_Heavy       EVQLVESGGGLVQPGGSLRLSCAASGFTVSSNYMSWVRQAPGKGLEWVSVIYSGGSTF  58
                                         ▪▪▪ ▪▪▪▪                  ▪▪▪▪▪▪▪
                ***********:**************. *****************************:

CV30_Heavy      YADSVKGRFTISRDNSKNTLYLQMNSLRAEDTAVYYCARDLDVSG-GMDVWGQGTTVTVS 117
                                                      ▪▪▪▪▪▪    ▪▪
B38_Heavy       YADSVKGRFTISRHNSKNTLYLQMNSLRAEDTAVYYCAREA---Y-GMDVWGQGTTVTVS 114
                                                      ▪▪▪   ▪   ▪▪
CB6_Heavy       YADSVKGRFTISRDNSMNTLFLQMNSLRAEDTAVYYCARVLPMYGDYLDYWGQGTLVTVS 118
                                                      ▪▪▪▪▪▪▪▪  ▪▪
                *************.** ***:******************        :* ***** ****


CV30_kappa      EIVLTQSPGTLSLSPGERATLSCRASQSVSSSYLAWYQQKPGQAPRLLIYGASSRATG  58
                                           ▪▪▪▪▪▪                       ▪
B38_kappa       DIVMTQSPSFLSASVGDRVTITCRASQGI-SSYLAWYQQKPGKAPKLLIYAASTLQSG  57
                                          ▪▪▪ ▪▪▪
CB6_kappa       DIVMTQSPSSLSASVGDRVTITCRASQSI-SRYLNWYQQKPGKAPKLLIYAASSLQSG  57
                                           ▪  ▪▪▪
                :**:****. ** * *:*.*::*****.: * ** *******:**:****.**:   :*

CV30_kappa      IPDRFSGSGSGTDFTLTISRLEPEDFAVYYCQQYGSS--PQTFGQGTKLEIKRTVAAPSV 116
                          ▪                       ▪▪
B38_kappa       VPSRFSGSGSGTEFTLTISSLQPEDFATYYCQQLNSY-PPYTFGQGTKLEIKRTVAAPSV 116
                         ▪▪▪                    ▪▪▪▪▪   ▪
CB6_kappa       VPSRFSGSGSGTDFTLTISSLQPEDFATYYCQQSYSTPPEYTFGQGTKLEIKRTVAAPSV 117
                         ▪                        ▪▪▪▪▪
                :*.*********:****** *:*****.*****  *     *******************
```

Supplementary Figure 2, related to Figure 2. Sequence alignment of CV30, B38, and CB6 heavy and light chain variable regions. Residues interacting with RBD are indicated with ▪. Conserved residues are indicated with * and similar residues are shown with : or . .

	CV30 Fab with SARS-CoV-2 RBD
**Data collection**	
Space group	$P6_3$
Cell dimensions	
a, *b*, *c* (Å)	147.44, 147.44, 89.38
α, β, γ (°)	90, 90, 120
Resolution (Å)	48.26 – 2.75 (2.85 – 2.75)
R_{merge}[a]	0.031 (0.4917)
<I/σ(I)>	13.01 (1.45)
$CC_{1/2}$	0.999 (0.573)
Completeness	99.93 (99.97)
Redundancy	2.0 (2.0)
**Refinement**	
Resolution (Å)	48.26 – 2.75 (2.85 – 2.75)
No. unique reflections	28886 (2862)
R_{work}[b]/R_{free}[c]	21.14/23.92 (32.83/35.35)
No. atoms	4917
Protein	4880
Water	23
Ligand	14
B-factors (Å^2)	78.69
Protein	78.70
Water	64.57
Ligand	98.31
RMS bond length (Å)	0.003
RMS bond angle (°)	0.63
**Ramachadran Plot Statistics**[d]	
Residues	638
Most Favored region	95.08
Allowed Region	4.60
Disallowed Region	0.32
Clashscore	**3.21**
**PDB ID**	**6XE1**

Supplementary Table 1, related to Figure 1. Data collection and refinement statistics for CV30 Fab with SARS-CoV-2 RBD.

[a] $R_{merge} = [\sum_h \sum_i |I_h - I_{hi}| / \sum_h \sum_i I_{hi}]$ where I_h is the mean of I_{hi} observations of reflection *h*. Numbers in parenthesis represent highest resolution shell. [b] R_{factor} and [c] $R_{free} = \sum||F_{obs}| - |F_{calc}|| / \sum|F_{obs}|$ x 100 for 95% of recorded data (R_{factor}) or 5% data (R_{free}). [d] Calculated using MolProbity (Williams et al. (2018) MolProbity: More and better reference data for improved all-atom structure validation. Protein Science 27: 293-315)

a.

SARS-CoV-2 RBD	HSDC	ASA	BSA
E:THR 415	H	100.21	36.26 ||||
E:GLY 416		21.15	16.96 |||||||||
E:LYS 417	H	111.60	64.06 ||||||
E:ASP 420	H	21.38	19.15 |||||||||
E:TYR 421	H	52.60	50.71 ||||||||||
E:TYR 453		40.70	20.50 ||||||
E:LEU 455	H	46.75	46.75 ||||||||||
E:PHE 456		64.15	57.70 |||||||||
E:ARG 457		49.01	6.19 ||
E:LYS 458	H	146.36	42.74 |||
E:SER 459		67.54	6.36 |
E:ASN 460		93.25	30.92 ||||
E:TYR 473	H	45.03	29.11 |||||||
E:GLN 474		63.55	3.44 |
E:ALA 475	H	56.82	56.33 ||||||||||
E:GLY 476		20.93	16.74 ||||||||
E:SER 477		110.57	29.70 |||
E:PHE 486		174.38	61.15 ||||
E:ASN 487	H	45.99	32.88 ||||||||
E:TYR 489	H	81.63	54.85 |||||||
E:GLN 493	H	75.64	29.87 ||||
E:SER 494		44.70	0.25 |

CV30 Heavy	HSDC	ASA	BSA
H:GLY 26		54.70	31.89 ||||||
H:VAL 27		10.36	8.54 |||||||||
H:ILE 28	H	91.56	48.86 ||||||
H:SER 30		26.64	2.94 ||
H:SER 31	H	71.94	61.25 |||||||||
H:ASN 32	H	23.41	21.21 ||||||||||
H:TYR 33	H	92.61	83.33 |||||||||
H:TYR 52	H	79.87	62.13 ||||||||
H:SER 53	H	48.87	40.00 |||||||||
H:GLY 54		81.91	57.87 ||||||||
H:SER 56	H	53.50	42.56 ||||||||
H:TYR 58	H	107.90	42.35 ||||
H:ARG 94	H	46.71	46.71 |||||||||||
H:ASP 95		34.02	1.23 |
H:LEU 96		53.68	47.15 |||||||||
H:ASP 97		93.71	42.36 |||||
H:VAL 98		146.30	50.39 ||||
H:SER 99	H	94.68	60.64 |||||||
H:ASP 101		72.77	9.56 ||
H:VAL 102		22.10	4.85 |||

b.

SARS-CoV-2 RBD	HSDC	ASA	BSA
E:TYR 453	H	40.70	20.20 |||||
E:PHE 486		174.38	7.35 |
E:SER 494		44.70	0.49 |
E:TYR 495		9.83	4.43 |||||
E:GLY 496		39.99	17.46 |||||
E:GLN 498		68.21	2.70 |
E:THR 500		125.50	7.00 |
E:ASN 501		38.08	15.54 |||||
E:GLY 502		39.16	25.23 |||||||
E:TYR 505	H	124.11	110.81 |||||||||

CV30 Light	HSDC	ASA	BSA
L:SER 27A		88.22	52.82 ||||||
L:VAL 28		8.77	7.19 |||||||||
L:SER 29		67.12	52.97 ||||||||
L:SER 30		45.62	2.21 |
L:SER 31		67.76	6.62 |
L:TYR 32	H	95.94	58.65 |||||||
L:THR 56		142.51	7.77 |
L:GLY 68		27.96	1.17 |
L:GLY 92		42.55	32.49 ||||||||
L:SER 93	H	47.50	24.38 ||||||

Supplementary Table 2, related to figure 1. Summary of interactions between SARS-CoV-2 RBD and CV30 Fab (from PISA web server, www.ebi.ac.uk). a. Detailed interactions of SARS-CoV-2 RBD and CV30 Fab Heavy Chain. **b.** Detailed interaction of SARS-CoV-2 RBD and CV30 Fab Light chain. HSDC notates a **H**ydrogen/**D**isulfide bond, **S**alt bridge or **C**ovalent link; Accessible Surface Area, (ASA, Å^2); Buried Surface Area (BSA, Å^2) with bars representing buried area percentage (one bar per 10%).

Ligand	Analyte	K_D (M X 10^{-9})	k_{on} (1/Ms) x10^5	k_{on} error x10^4	k_{off} (1/s) x10^{-2}	K_{off} error x10^{-3}
glCV30 IgG	SARS-CoV-2 RBD	407	1.83	1.40	7.46	1.12

Supplementary Table 3, related to Figure 1. Kinetic analysis of glCV30 binding to SARS-CoV-2 RBD. Kinetic values were calculated using a 1:1 binding model. Data was collected from two independently prepared analyte dilution series. K_D values were determined from the mean values of k_{on} and k_{off} that matched the theoretical fit with an R^2 value of ≥0.98
