# Supplementary material for: Structural basis for potent neutralization of SARS-CoV-2 and role of antibody affinity maturation
Source: Nat Commun. 2020 Oct 27;11:5413. doi: 10.1038/s41467-020-19231-9 (PMC7591918; doi:10.1038/s41467-020-19231-9)
Supplement: Supplementary file 3 — Reporting Summary [file 41467_2020_19231_MOESM3_ESM.pdf]

nature research

Corresponding author(s): Marie Pancera

Last updated by author(s): Sept 18th , 2020

Reporting Summary

Nature Research wishes to improve the reproducibility of the work that we publish. This form provides structure for consistency and transparency in reporting. For further information on Nature Research policies, see our Editorial Policies and the Editorial Policy Checklist.

Statistics

For all statistical analyses, confirm that the following items are present in the figure legend, table legend, main text, or Methods section.

n/a	Confirmed	
☒	☐	The exact sample size (*n*) for each experimental group/condition, given as a discrete number and unit of measurement
☒	☐	A statement on whether measurements were taken from distinct samples or whether the same sample was measured repeatedly
☒	☐	The statistical test(s) used AND whether they are one- or two-sided<br>*Only common tests should be described solely by name; describe more complex techniques in the Methods section.*
☒	☐	A description of all covariates tested
☒	☐	A description of any assumptions or corrections, such as tests of normality and adjustment for multiple comparisons
☐	☒	A full description of the statistical parameters including central tendency (e.g. means) or other basic estimates (e.g. regression coefficient) AND variation (e.g. standard deviation) or associated estimates of uncertainty (e.g. confidence intervals)
☒	☐	For null hypothesis testing, the test statistic (e.g. *F*, *t*, *r*) with confidence intervals, effect sizes, degrees of freedom and *P* value noted<br>*Give P values as exact values whenever suitable.*
☒	☐	For Bayesian analysis, information on the choice of priors and Markov chain Monte Carlo settings
☒	☐	For hierarchical and complex designs, identification of the appropriate level for tests and full reporting of outcomes
☒	☐	Estimates of effect sizes (e.g. Cohen's *d*, Pearson's *r*), indicating how they were calculated

Our web collection on statistics for biologists contains articles on many of the points above.

Software and code

Policy information about availability of computer code

Data collection	XDS (diffraction data processing), ForteBio Octet Data Acquisition 9.0
Data analysis	CCP4 7.1.002, Aimless 0.7.4, Phenix v1.17.1, Phaser v 2.8.3, WinCOOT 0.8.9.2, PyMol 1.8.2.0, ForteBio Octet Data Analysis 9.0 Octet, GraphPad Prism 8.4

For manuscripts utilizing custom algorithms or software that are central to the research but not yet described in published literature, software must be made available to editors and reviewers. We strongly encourage code deposition in a community repository (e.g. GitHub). See the Nature Research guidelines for submitting code & software for further information.

Data

Policy information about availability of data

All manuscripts must include a data availability statement. This statement should provide the following information, where applicable:

- Accession codes, unique identifiers, or web links for publicly available datasets
- A list of figures that have associated raw data
- A description of any restrictions on data availability

Coordinates and structure factors for CV30 Fab-SARS-CoV-2 RBD complex have been deposited in the Protein Data Bank (PDB) under the accession code 6XE1. [[https://www.rcsb.org/structure/6XE1]. Additionally these publicly available datasets are mentioned. https://www.rcsb.org/structure/6LZG]), (PDB: 7BZ5 [https://www.rcsb.org/structure/7BZ5]), (PDB: 7C01 [https://www.rcsb.org/structure/7C01]), PDBid: 5I1E {[https://www.rcsb.org/structure/5I1E]

Field-specific reporting

Please select the one below that is the best fit for your research. If you are not sure, read the appropriate sections before making your selection.

☒ Life sciences ☐ Behavioural & social sciences ☐ Ecological, evolutionary & environmental sciences

For a reference copy of the document with all sections, see nature.com/documents/nr-reporting-summary-flat.pdf

Life sciences study design

All studies must disclose on these points even when the disclosure is negative.

Sample size	We did not perform sample size calculation as our aim was to obtain a structure of a known neutralizing antibody, CV30 in complex with its epitope. We also performed additional experiments to understand the mechanism of neutralization.
Data exclusions	No data were excluded from the analyses
Replication	Binding and neutralization experiments were performed in duplicate. All attempts at replications were successful. For the structure, this was done once and proper validation confirmed the validity of the structure.
Randomization	No randomization was applicable to the study as we wanted to assess the structural and biological mechanism of known antibodies.
Blinding	No blinding was applicable to the study as we specifically studied known antibodies.

Reporting for specific materials, systems and methods

We require information from authors about some types of materials, experimental systems and methods used in many studies. Here, indicate whether each material, system or method listed is relevant to your study. If you are not sure if a list item applies to your research, read the appropriate section before selecting a response.

Materials & experimental systems

n/a	Involved in the study	
☐	☒	Antibodies
☐	☒	Eukaryotic cell lines
☒	☐	Palaeontology and archaeology
☒	☐	Animals and other organisms
☒	☐	Human research participants
☒	☐	Clinical data
☒	☐	Dual use research of concern

Methods

n/a	Involved in the study	
☒	☐	ChIP-seq
☐	X	Flow cytometry
☒	☐	MRI-based neuroimaging

Antibodies

Antibodies used	CV30 and glCV30 Antibody and Antigen binding fragment, prepared in house; secondary antibody Goat anti-Human Ig-HRP (Southern Biotech, Cat# 2010-05) ; PE-conjugated AffiniPure Fab fragment goat anti-human IgG (1:50 dilution, Jackson Immunoresearch, Cat #109-117-008)
Validation	For the commercially available antibodies, certificate of analysis are available at manufacturer website to provide validation. For antibodies developed in this study such as CV30 and glCV30, validation was done by binding and neutralization experiments as well as structural information.

Eukaryotic cell lines

Policy information about cell lines

Cell line source(s)	HEK293T, ATCC, Cat# CRL-3216; 293SGD (see reference); HEK293T-hACE2, BEI resources, Cat# NR-5251; 293-6E, National Research Council of Canada (under license), no cat #; VeroE6 cell (C1008, ATCC, #CRL-1586)
Authentication	STR profiling
Mycoplasma contamination	Mycoplasma tests were performed using the MycoProbe kit from R and D Systems and the samples were negative for contamination.
Commonly misidentified lines (See ICLAC register)	No commonly misidentified cell lines were used in this study
